# Clinical and genetic characteristics of 14 patients from 13 Japanese families with *RPGR*-associated retinal disorder: report of eight novel variants

**DOI:** 10.1038/s41439-019-0065-7

**Published:** 2019-08-02

**Authors:** Go Mawatari, Kaoru Fujinami, Xiao Liu, Lizhu Yang, Yu-Fujinami Yokokawa, Shiori Komori, Shinji Ueno, Hiroko Terasaki, Satoshi Katagiri, Takaaki Hayashi, Kazuki Kuniyoshi, Yozo Miyake, Kazushige Tsunoda, Kazutoshi Yoshitake, Takeshi Iwata, Nobuhisa Nao-i, on behalf of the JEGC study group

**Affiliations:** 1 Department of Ophthalmology, Faculty of Medicine, University of Miyazaki, Kiyotake, Miyazaki, Japan; 2 Laboratory of Visual Physiology, Division of Vision Research, National Institute of Sensory Organs, National Hospital Organization Tokyo Medical Center, Meguro-ku, Tokyo, Japan; 3 Department of Ophthalmology, Keio University School of Medicine, Tokyo, Japan; 4 UCL Institute of Ophthalmology, London, UK; 5 Moorfields Eye Hospital, London, UK; 6 Southwest Hospital/Southwest Eye Hospital, Third Military Medical University, Chongqing, China; 7 Graduate School of Health Management, Keio University, Tokyo, Japan; 8 Division of Public Health, Yokokawa Clinic, Suita, Osaka, Japan; 9 Department of Ophthalmology, Nagoya University Graduate School of Medicine, Showa-ku, Nagoya, Aichi Japan; 10 Department of Ophthalmology, The Jikei University School of Medicine, Nishi-Shimbashi, Minato-ku, Tokyo, Japan; 11 Department of Ophthalmology, Kinki University Faculty of Medicine, Osaka-Sayama City, Osaka, Japan; 12 Kobe Eye Center, Next Vision, Kobe, Hyogo, Japan; 13 Division of Molecular and Cellular Biology, National Institute of Sensory Organs, National Hospital Organization Tokyo Medical Center, Meguro-ku, Tokyo, Japan

**Keywords:** Medical genetics, Genetic predisposition to disease

## Abstract

Variants in the retinitis pigmentosa GTPase regulator (RPGR) gene are a major cause of X-linked inherited retinal disorder (IRD). We herein describe the clinical and genetic features of 14 patients from 13 Japanese families harboring *RPGR* variants in a nationwide cohort. Comprehensive ophthalmological examinations were performed to classify the patients into one of the phenotype subgroups: retinitis pigmentosa (RP) and cone rod dystrophy (CORD). The mean age of onset/at examination was 13.8/38.1 years (range, 0–50/11–72), respectively. The mean visual acuity in the right/left eye was 0.43/0.43 (range, 0.1–1.7/−0.08–1.52) LogMAR unit. Eight patients had RP, and six had CORD. Whole-exome sequencing with target analyses identified 13 *RPGR* variants in 730 families with IRD, including 8 novel variants. An association between the phenotype subgroup and the position of variants (cutoff of amino acid 950) was revealed. To conclude, the clinical and genetic spectrum of *RPGR*-associated retinal disorder was first illustrated in a Japanese population, with a high proportion of novel variants. These results suggest the distinct genetic background of RPGR in the Japanese population, in which the genotype–phenotype association was affirmed. This evidence should be helpful monitoring and counseling patients and in selecting patients for future therapeutic trials.

## Retinal disorders: Seeing new patterns

New mutations that cause inherited retinal disorders (IRD) such as retinitis pigmentosa have been identified in Japanese patients. IRD, which affect the light-sensing rods and cones of the eye, often appear in childhood, and may cause blindness by middle age. IRD have been traced to mutations of the retinal development gene *RPGR*, but have mainly been studied in European populations. Go Mawatari and coworkers studied the genetics of IRD in 14 Japanese patients, and searched genetic data collected during the past decade from over 1000 Japanese patients with IRD registered in the Japan Eye Genetics Consortium database. They found eight novel *RPGR* mutations and noticed that mutations in different parts of the gene cause different types of IRD. These data will help in diagnosis and counselling of patients with these rare eye disorders.

## Introduction

Inherited retinal disorder (IRD) is a major cause of blindness both in children and working populations in developed countries^1^. Retinitis pigmentosa (RP) is one of the most prevalent IRDs, and RP represents a heterogeneous group of retinal diseases characterized by progressive bilateral degeneration of rod and cone photoreceptors^1–8^. The estimated prevalence of RP in European populations is ~1 in 3000–4000 individuals^2–6,9^. Different patterns of inheritance have been identified in RP and allied disorders, including autosomal dominant (AD), autosomal recessive (AR), X-linked (XL), and mitochondrial inheritance^2,3,10^.

RP with an X-linked pattern of inheritance (XLRP) accounts for ~10–15% of RP cases, and is associated with the most severe form of the disease^3,5,7,8,11^. Two major causative genes for XLRP are the retinitis pigmentosa GTPase regulator (RPGR; OMIM; 312610) and RP2 (OMIM; 312600), which accounts for 70–90% and 7–18% of XLRP, respectively^9^.

Pathogenic variants in the *RPGR* gene (RP3) were first identified as a cause of XLRP in 1996^12,13^. *RPGR* contains 19 exons and encodes a 90-kDa protein product localized predominantly to the photoreceptor connecting cilium (CC)^12,14^. The RPGR protein contains a repeat structure highly similar to the regulator of chromosome condensation 1 (RCC1) at the N-terminus^11,12,15^. RCC1 plays a crucial role in nucleocytoplasmic transport and regulation of cell-division processing^16,17^. Later, a novel 3′ terminal exon (well-known as exon open-reading frame 15; ORF15) was identified, which includes a large 3′ terminal exon consisting of exon 15 and extending into part of intron 15^18^. Biochemical investigations revealed that RPGR-ORF15 is located in the CC, which binds to the axoneme and the basal body^19,20^. The RPGR protein plays an important role in the transportation of phototransduction components and other outer segment proteins across the CC, although the function of RPGR is not perfectly understood^3^.

XLRP caused by pathogenic *RPGR* variants is one of the most severe forms of RP, with early onset of disease, night blindness, myopia, severe generalized rod and cone dysfunction, and progression to legal blindness by the third or fourth decade^3,21^. Carrier females are mostly asymptomatic or mildly affected with characteristic fundus features and electrophysiological abnormalities^22^, although the severity of carriers varies.

Pathogenic variants in the RPGR gene were responsible for X-linked cone rod dystrophy (XLCORD) and XL cone dystrophy (XLCOD), in addition to XLRP^23–28^. *RPGR-*associated retinal disorder (*RPGR*-RD) accounts for 73% of molecularly confirmed XLCORD cases in a British cohort. *RPGR* variants identified in XLCORD/XLCOD are frequently located toward the 3′ end of ORF15 in comparison with *RPGR* variants causing XLRP^3,29,30^.

XLCORD caused by pathogenic *RPGR* variants affects males with various onsets ranging from the second to the fourth decade, myopia, generalized cone rod dysfunction (occasionally with rod dysfunction), and diverse rates of progression^3^. Carrier females are mostly asymptomatic or mildly affected, with varying severity^28^.

Over 350 disease-associated variants in the *RPGR* gene have been reported to date in IRD^3,15,26,31–37^. A number of studies have been conducted in European populations;^3,11,21,27,37–41^ however, the characteristics of *RPGR*-RD in Asian populations remain uncertain due to limited resources^8,34–36,41^. Therefore, large cohort studies are required to understand the *RPGR*-RD in Asian populations.

The purpose of this study was to characterize the clinical and genetic features of patients and carriers with *RPGR*-RD in a large nationwide Japanese cohort by clarifying a genotype–phenotype association.

## Methods

### Participants

The protocol of this study adhered to the tenets of the Declaration of Helsinki, which was approved by the local ethics committee of the participating institutions from Japan (Reference: R18-029). Signed informed consent was obtained from all participants after explanation of the nature and possible consequences of this study.

Patients with a clinical diagnosis of IRD and available genetic data of whole-exome sequencing (WES) were investigated between 2008 and 2018 in the Japan Eye Genetics Consortium (JEGC; http://www.jegc.org/) study^42^. A total of 1294 subjects from 730 Japanese families registered to the JEGC database were surveyed.

### Clinical investigations

Detailed demographic information was obtained, including ethnicity, sex, medical and family history, chief complaints of visual symptoms, and onset of disease. Comprehensive ophthalmological examinations were performed, including measurement of refractive errors, best corrected decimal visual acuity (BCVA) converted to the logarithm of the minimum angle of resolution (LogMAR), fundus photography, fundus autofluorescence (FAF) imaging, spectral-domain optical coherence tomography (SD-OCT), visual field testing, and electrophysiological assessment according to the international standards of the International Society for Clinical Electrophysiology of Vision (ISCEV)^43,44^.

### Phenotype subgroup

For the purpose of this study, phenotype subgroups were defined based on clinical manifestation according to the previous report;^45^ RP (including rod-cone dystrophy), a progressive retinal dystrophy initially often presenting peripheral atrophy with generalized rod dysfunction greater than cone dysfunction; CORD, a progressive retinal dystrophy initially often presenting with macular atrophy with generalized cone dysfunction greater than rod dysfunction.

### *RPGR* variant detection

Genomic DNA was extracted from all affected subjects and unaffected family members (where available for cosegregation analysis). WES with target analysis of retinal disease-associated genes (RetNet; https://sph.uth.edu/retnet/home.htm) was performed according to previously published methods^42^. The called variants were filtered by the allele frequency in the general Japanese population (<1%) as listed in the Human Genetic Variation Database (HGVD; http://www.genome.med.kyoto-u.ac.jp/SnpDB/about.htm). Depth and coverage for the target areas were interrogated using the Integrative Genomics Viewer (http://www.broadinstitute.org/igv/). For the purpose of this study, long-read direct sequencing was performed in seven patients with XLRP who were negative for two major XLRP-associated genes (RP2, RPGR) by WES at the National Genetic Reference Laboratory in Manchester, UK, for further screening of RPGR-ORF15 according to the previously published method^46^. Together with clinical features and pattern of inheritance, disease-causing variants were determined from the detected variants of the retinal disease-associated genes.

### In silico molecular genetic analysis

The allele frequencies of all detected *RPGR* variants were established with the HGVD, Integrative Japanese Genome Variation (iJGVD 3.5k; https://ijgvd.megabank.tohoku.ac.jp/download_3.5kjpn/), 1000 Genomes (http://www.internationalgenome.org/), and the Genome Aggregation Database (gnomAD; http://gnomad.broadinstitute.org/). All detected *RPGR* variants were analyzed with two general and three functional prediction programs; MutationTaster (http://www.mutationtaster.org/), FATHMM (http://fathmm.biocompute.org.uk/9), SIFT (https://www.sift.co.uk/), PROVEAN (http://provean.jcvi.org/index.php), and Polyphen 2 (http://genetics.bwh.harvard.edu/pph2/). All detected *RPGR* variants were analyzed with evolutionary conservation scores according to the UCSC database (https://genome.ucsc.edu/index.html). Variant classification according to the guidelines of the American College of Medical Genetics and Genomics (ACMG) was conducted for all detected variants^47^.

## Results

### Participants

Fourteen affected subjects from 13 Japanese families with a clinical diagnosis of IRD and harboring RPGR variants were ascertained. All 14 affected subjects were registered as a proband (or probands) for each pedigree. Seven females from six families were also registered as carriers.

**Fig. 1:**
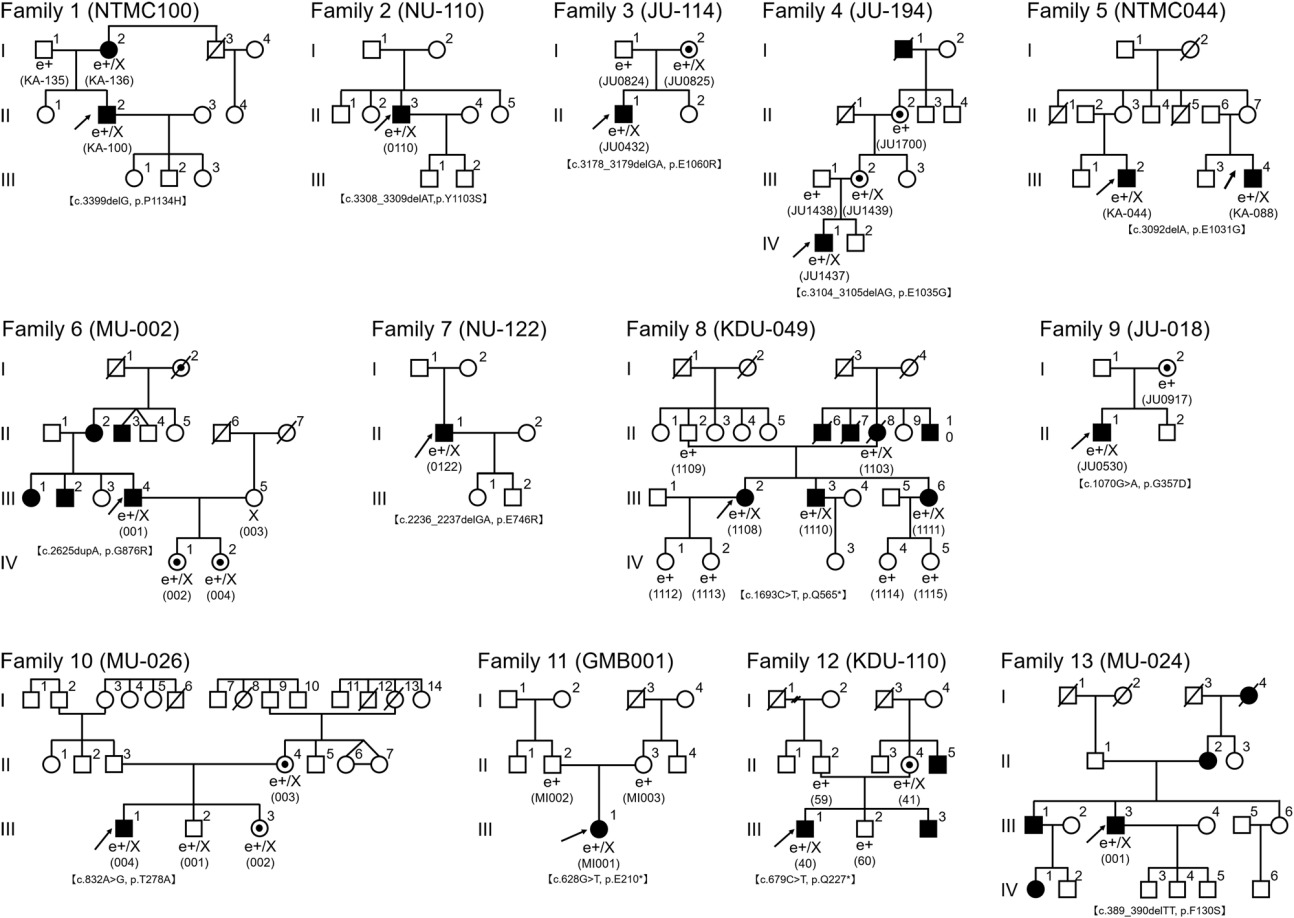
Pedigrees of 13 Japanese families with *RPGR*-associated retinal disorder (*RPGR*-RD). The solid squares (men) and circles (women) represent the affected patients. Carrier females are represented by circles containing a black spot. Unaffected family members are represented by white icons. The slash symbol indicates deceased individuals. The generation number is shown on the left. The proband of each pedigree is marked by an arrow. Subjects clinically examined and genetically examined are annotated with e + and X

The detailed clinical information of 14 affected subjects (registered as a proband) is presented in Table 1. Pedigrees of 13 families are shown in Fig. 1. All 14 subjects and 7 carriers were originally from Japan, and no mixture of ethnicity was reported.

**Table 1:** Clinical features of 14 Japanese patients with *RPGR*-associated retinal disorder

Family no.	Patient no.	Inheritance	Sex	Age (in the database)	Age (at latest examination)	Onset	Chief complaint	LogMAR BCVA		Spherical equivalent		Fundus/AF findings	OCT findings	Visual field	Full-field ERG	Phenotype
								RE	LE	RE	LE					
1	1-II:2	Possible XL/AD	M	57	54	50	Photophobia	0.4	−0.08	−1.00 (post LASIK)	−1.00 (post LASIK)	Central retinal atrophy/central hypo AF surrounded by hyper AF ring	Outer retinal atrophy at the central retina	Central scotoma (GP)	Severely decreased cone reponses and mildly decreased rod responses	Cone rod dystrophy
2	2-II:3	Sporadic	M	74	72	NA	Poor visual acuity	1.7	1.52	+ 0.50 (cataract)	−0.50 (cataract)	Central retinal atrophy/ attenuated blood vessels	Outer retinal atrophy at the central retina	Central scotoma and concentric visual field defect (GP)	Underctable cone and rod responses	Cone rod dystrophy
3	3-II:1	Sporadic	M	50	50	15	Reduced visual acuity	0.7	0.82	−7.00	−6.50	Tigroid/central retinal atrophy/central hypo AF surrounded by hyper AF ring	Outer retinal atrophy at the central retina	Central scotoma (GP)	Severely decreased cone responses and mildly decreased rod responses	Cone rod dystrophy
4	4-IV:1	Definite XL	M	11	11	4	Reduced visual acuity	0.15	0.1	−4.00	−4.50	Tigroid/paracentral hyper AF/hyper AF ring	Outer retinal atrophy at the paramacula	No particular scotoma (GP)	Moderately decreased cone and rod responses	Cone rod dystrophy
5	5-III:2	Possible XL/incomplete AD	M	50	47	30	Color vision abnormality	0.22	0.22	−4.00	−4.00	Tigroid/ born spicule pigmentosa, central and paracentral retinal atrophy/hyper AF ring	Outer retinal atrophy at the central retina	Central scotoma (GP)	Severely decreased cone and mildly decreased rod responses	Cone rod dystrophy
5	5-III:4	Possible XL/incomplete AD	M	47	44	15	Photophobia	0.3	1.1	−8.00	−7.00	Tigroid/ centeral and paracentral retinal atrophy/hyper AF ring	Outer retinal atrophy at the central retina	Central scotoma (GP)	Severely decreased cone and rod responses	Cone rod dystrophy
6	6-III:4	Possible XL/AD	M	45	41	8	Reduced visual acuity	0.82	0.7	−7.00	−6.00	Tigroid/ paracentral retinal atrophy/ born spicule pigmentation/attenuated blood vessels	Outer retinal atrophy at the central retina、thinning choroid	Concentric visual field defect (GP)	Undetectable cone and rod responeses	Retinitis pigmentosa
7	7-II:1	Sporadic	M	52	50	NA	Poor visual acuity	0.52	0.52	+ 1.00	+ 1.00	Tigroid/ paracentral retinal atrophy/born spicule pigmentation/attenuated blood vessels	Outer retinal atrophy at the paracentral retina, thinning choroid	Concentric visual field defect (GP)	NA	Retinitis pigmentosa
8	8-III:2	Possible XL/AD	F	50	41	NA	Night blindness	0.1	0.15	−10	−13.5	NA	NA	No particular scotoma (GP)	Moderately decreased cone and rod responses	Retinitis pigmentosa
9	9-II:1	Probable XL	M	33	32	5	Night blindness	0.1	0	0	+ 0.50	Paracentral retinal atrophy/born spicule pigmentation/ attenuated blood vessels	Outer retinal atrophy at the paracentral retina	Annular scotoma (GP)	Undetectable cone and rod responeses	Retinitis pigmentosa
10	10-III:1	Probable XL	M	17	17	12	Night blindness	0.3	−0.18	−1.50	−1.00	Paracentral retinal atrophy/hyper AF ring	Outer retinal atrophy at the paracentral retina	Partial paracentral scotoma (GP)	Severely decreased rod responses and moderately decreased cone responses	Retinitis pigmentosa
11	11-III:1	Sporadic	F	25	25	9	NA	0.15	0.15	−6.50	−7.00	NA	Outer retinal atrophy at the paracentral retina、thinning choroid	Partial paracentral scotoma (GP)	Undetectable rod and cone responeses	Retinitis pigmentosa
12	12-III:1	Definite XL	M	16	14	0	Night blindness	0.4	0.3	0	−1.00	Paracentral retinal atrophy	Outer retinal atrophy at the paracentral retina、thinning choroid	Annular scotoma (GP)	NA	Retinitis pigmentosa
13	13-III:3	Possible XL/AD	M	36	35	4	Peripheral visual field loss	0.22	0.1	−1.00	−1.00	Paracentral retinal atrophy/born spicule pigmentation/attenuated blood vessels	Outer retinal atrophy at the paracentral retina	Annular scotoma (GP)	Undetectable rod and cone responeses	Retinitis pigmentosa

*AD* autosomal dominant, *LogMAR BCVA* best-corrected visual acuity, *CS* central scotoma, *F* female, *FS* foveal sparing, *LE* left eye, *M* male, *RE* right eye, *NA* not available, *OCT* spectral-domain optical coherence tomography, *AF* autofluorescence, *GP* Goldmann Perimetry, *HFA* Humphrey visual field analyzer, *LASIK* laser in situ keratomileusis, *ERG* electroretinogram

Age was defined the age when the latest examination was performed. The age of onset was defined as either the age at which visual loss was first noted by the patient or, in the “asymptomatic” patients, when an abnormal retinal finding was first detected

There were two families with definite XL family history (2/13, 15.4%; Families 4, 12; history of multiple affected males in different generations and a female carrier for at least one generation), two families with probable XL history (2/13, 15.4%; Families 9, 10, one affected male and at least a female carrier), five families with possible XL/AD/incomplete AD (5/13, 38.5%; Families 1, 5, 6, 8, 13; a transmission between at least two generations reported or an incomplete transmission anticipated), and three with unknown family history (4/13, 30.8%, Families 2, 3, 7, 11; sporadic).

There were 12 affected males (12/14, 85.7%) and 2 affected females (2/14, 14.2%). For the purpose of this study, the two affected females registered as probands are described as patients, since both had clear visual impairment (8-III:2, 12-III:1). Systemic abnormalities, including hearing loss, were not reported in all patients.

The mean age at the latest examination of 12 affected males and 2 affected females was 38.9 (range, 11–72) and 25.0 years (25, 41), respectively.

### Onset, chief complaint, refraction, and visual acuity

The mean age of onset of ten affected males with available records was 14.3 years (range, 0–50). One affected female with available records had onset of disease at the age of 9 (11-III:1).

Night blindness was reported in 4 out of 11 patients with available records (4/11, 36.3%). There were three patients with reduced visual acuity (3-II:1, 4-IV:1, 6-III:4), two with poor visual acuity (2-II:3, 7-II:1), two with photophobia (1-II:2, 5-III:4), one with color vision abnormality (5-III:2), and one with peripheral visual field defect (13-III:3).

The mean refractive error of the right/left eye of ten affected males with available records who had no refractive complication was −3.15/−2.95 diopter (range −8.0–1.0/−7.0–1.0). The mean refractive error of the right/left eye of two affected females with available records was −8.25/−10.25 diopter (−10, −6.5/−13.5, −7.0). One patient had cataracts in both eyes (2-II:3), and one patient underwent refractive surgery for myopia (1-II:2). Five patients had high myopia (5/10, 50.0%; higher than −6.0 diopter).

The mean VA in the right/left eye of ten affected males was 0.49/0.48 LogMAR unit (range 0.1–1.7/−0.08–1.52). The mean VA in the right/left eye of two affected females was 0.13/0.15 (0.1, 0.15/0.15, 0.15) LogMAR unit. Eight patients had relatively favorable VA (8/14, 57.1%; 0.22 LogMAR unit or better in the better eye), five had moderate VA (5/14, 35.7%; between 0.22 and 1.0 LogMAR unit in the better eye), and one had poor VA (1/14, 7.1%; 1.0 LogMAR unit or worse in the better eye).

### Retinal images, visual field, and electrophysiological findings

**Fig. 2:**
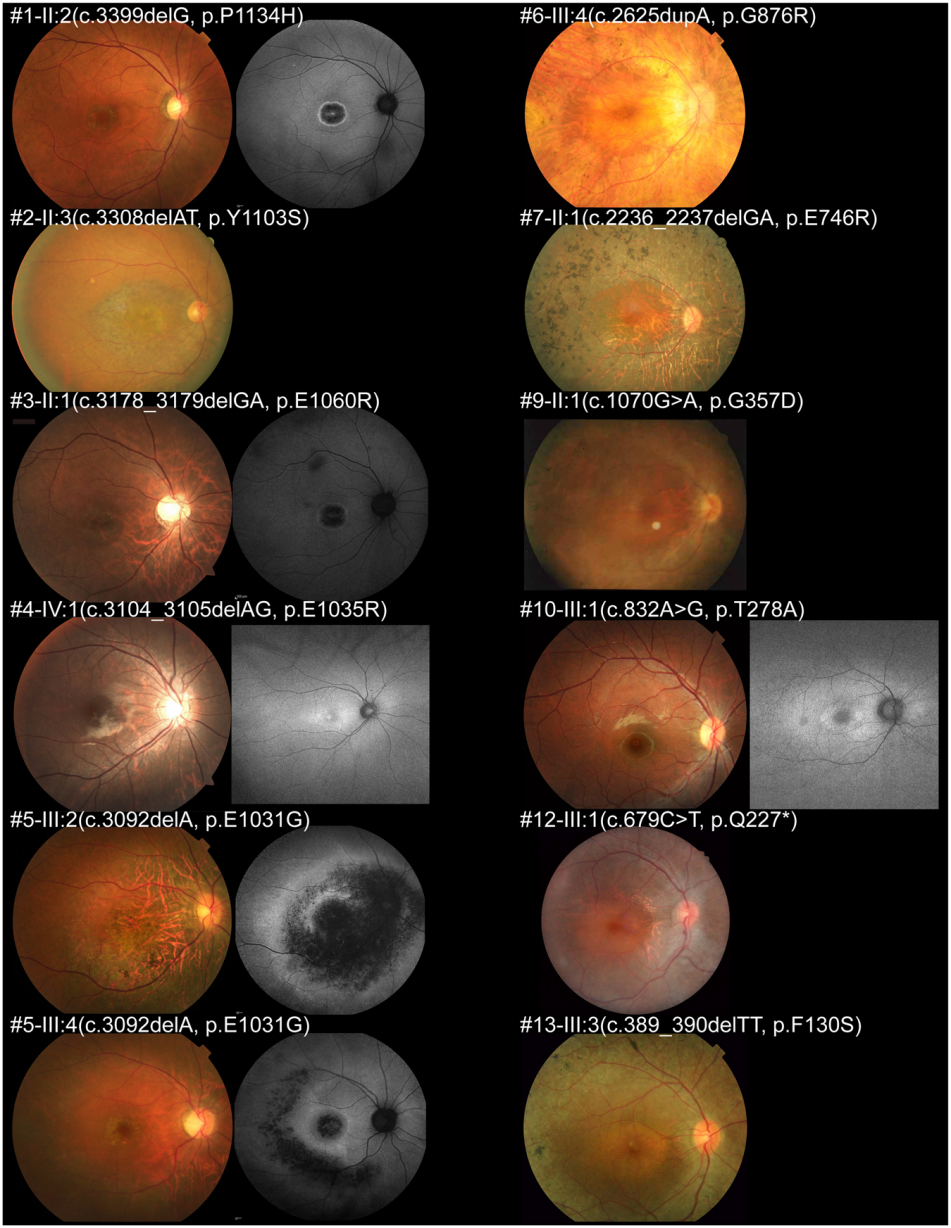
Fundus photographs and fundus autofluorescence images of 12 patients with *RPGR*-RD. Fundus photographs and autofluorescence (FAF) images of the right eyes of 12 affected males with *RPGR*-RD are presented. Central atrophy or parafoveal atrophy was identified in all patients, with tigroid changes (seen in high myopic retina) in six patients (3-II:1, 4-IV:1, 5-III:2, 5-III:4, 6-III:4, and 7-II:1), and bone-spicule pigmentation (found in peripheral retinal atrophy) in five patients (5-III:2, 6-III:4, 7-II:1, 9-II:1, and 13-III:3). Well-marked atrophic changes with a ring of high AF density were found in all patients with available FAF images

Fundus photographs were available in 12 affected males, and FAF images were obtained in 6 affected males. Representative fundus and FAF images of 12 affected males are presented in Fig. 2.

Central atrophy or parafoveal atrophy was identified in all 12 patients with available fundus photographs. Tigroid changes (seen in high myopic retina) were observed in six patients (6/12, 50%), and bone-spicule pigmentation (found in peripheral retinal atrophy) was detected in five patients (5/12, 41.7%). Well-marked atrophic changes were demonstrated in FAF images. A ring of high AF density was noted in all six patients with available FAF images.

**Fig. 3:**
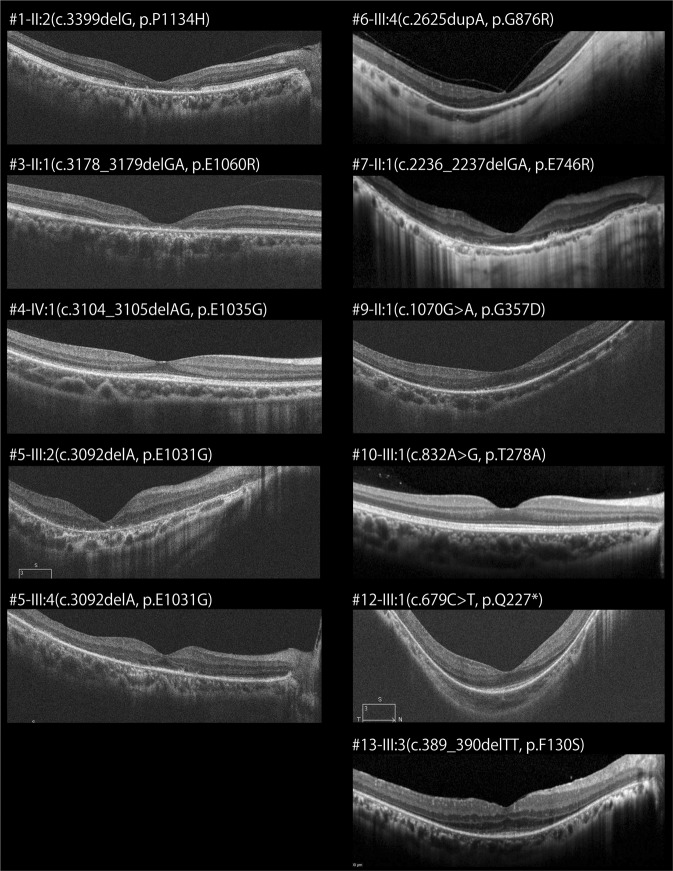
Spectral-domain optical coherence tomography of 11 patients with *RPGR*-RD. Spectral-domain optical coherence tomographic images of the right eyes of 11 affected males are presented. Structural disruption in the photoreceptor layers is observed in all patients, with relatively preserved photoreceptor layers at the fovea in five patients (4-IV:1, 7-II:1, 10-III:1, 12-III:1, and 13-III:3)

SD-OCT images were available in 11 affected males. Representative SD-OCT images are shown in Fig. 3. Structural disruption in the photoreceptor layers was observed in all 11 patients. Relatively preserved photoreceptor layers at the fovea were detected in five patients (5/11, 45.5%; 4-IV:1, 7-II:1, 10-III:1, 12-III:1, and 13-III:3).

Visual fields were available in all 12 affected males and 2 affected females. Central scotoma was observed in four patients (4/14, 28.6%), and annular scotoma was detected in three patients (3/14, 21.4%). Concentric visual field defects were found in two patients (2/14, 14.3%), and both central scotomas and concentric defects were observed in one patient (1/14, 7.1%). Two patients had partial paracentral scotoma (2/14, 14.3%), and two had no particular visual field defect (2/14, 14.3%).

Electrophysiological assessment was performed in ten affected males and two affected females. Undetectable responses in both generalized rod and cone systems were observed in five patients (5/12, 41.7%). Severely decreased generalized cone and severely decreased rod responses were demonstrated in one patient (1/12, 8.3%). Severely decreased generalized rod responses and moderately decreased generalized cone responses were identified in one patient (1/12, 8.3%). Severely decreased generalized cone and mildly decreased generalized rod responses were found in three patients (3/12, 25.0%). Moderately decreased generalized cone and generalized rod responses were noted in two patients (2/12, 16.7%).

### Phenotype subgroups

Phenotype subgroup classification was performed in all 12 affected males and 2 affected females. There were six males with CORD (6/12, 50%) and six with RP (6/12, 50%). Both affected females were classified into RP.

### Clinical information of carrier females

Clinical information of seven carrier females from six families was obtained. The detailed findings are described in Supplementary Table 1. Representative fundus and FAF images are presented in Supplementary Fig. 1. Representative SD-OCT images are shown in Supplementary Fig. 2.

The mean age at the latest examination of seven carriers was 47.1 years (range, 12–78). The mean refractive error of the right/left eye was −7.00/−7.42 diopter (range, −12.5–0.5/−12.0–0.5). The mean BCVA in the right/left eye was 0.14/0.13 LogMAR unit (range, 0.05–0.4/0.0–0.4).

Abnormalities on fundus photographs, FAF, SD-OCT, visual field testing, and electrophysiological assessment were found in five out of seven carriers (5/7, 71.4%). One carrier had reduced visual acuity, and one had night blindness. Retinal atrophy was observed in two carriers, and tapetal reflexes were found in five carriers (5/7, 71.4%). Electrophysiological assessment was available in five carriers, and all five carriers showed decreased responses.

### *RPGR* variants

The variant data of 18 affected, 9 carriers, and 14 unaffected subjects of 13 families are summarized in Table 2.

**Table 2:** Summary of detected variants of 18 affected, 9 carriers, and 14 unaffected individuals from 13 families with RPGR-associated retinal disorder

Family no.	Patient no.	Gender	Affected/unaffected	Exon	Nucleotide and amino acid changes	State
1	1-II:2	Male	Affected	15	*c.3399delG, p.Pro1134HisfsTer18*	Hemizygous
	1-I:1	Male	Unaffected		ND	
	1-I:2	Female	Affected	15	*c.3399delG, p.Pro1134HisfsTer18*	Heterozygous
2	2-II:3	Male	Affected	15	c.3308_3309delAT, p.Tyr1103SerfsTer7	Hemizygous
3	3-II:1	Male	Affected	15	c.3178_3179delGA, p.Glu1060ArgfsTer18	Hemizygous
	3-I:1	Male	Unaffected		ND	
	3-I:2	Female	Carrier	15	c.3178_3179delGA, p.Glu1060ArgfsTer18	Heterozygous
4	4-IV:1	Male	Affected	15	*c.3104_3105delAG, p.Glu1035GlyfsTer43*	Hemizygous
	4-III:1	Male	Unaffected		ND	
	4-III:2	Female	Carrier	15	*c.3104_3105delAG, p.Glu1035GlyfsTer43*	Heterozygous
	4-II:2	Female	Carrier		ND	
5	5-III:2	Male	Affected	15	c.3092delA, p.Glu1031GlyfsTer58	Hemizygous
	5-III:4	Male	Affected	15	c.3092delA, p.Glu1031GlyfsTer58	Hemizygous
6	6-III:4	Male	Affected	15	c.2625dupA, p.Gly876ArgfsTer203	Hemizygous
	6-IV:1	Female	Carrier	15	c.2625dupA, p.Gly876ArgfsTer203	Heterozygous
	6-III:5	Female	Unaffected		ND	
	6-IV:2	Female	Carrier	15	c.2625dupA, p.Gly876ArgfsTer203	Heterozygous
7	7-II:1	Male	Affected	15	c.2236_2237delGA, p.Glu746ArgfsTer23	Hemizygous
8	8-III:2	Female	Affected	14	*c.1693C* *>* *T, p.Gln565Ter*	Heterozygous
	8-II:8	Female	Affected	14	*c.1693C* *>* *T, p.Gln565Ter*	Heterozygous
	8-II:2	Male	Unaffected		ND	
	8-III:3	Male	Affected	14	*c.1693C* *>* *T, p.Gln565Ter*	Hemizygous
	8-III:6	Female	Affected	14	*c.1693C* *>* *T, p.Gln565Ter*	Heterozygous
	8-IV:1	Female	Unaffected		ND	
	8-IV:2	Female	Unaffected		ND	
	8-IV:4	Female	Unaffected		ND	
	8-IV:5	Female	Unaffected		ND	
9	9-II:1	Male	Affected	10	*c.1070* *G* *>* *A, p.Gly357Asp*	Hemizygous
	9-I:2	Female	Carrier		ND	
10	10-III:1	Male	Affected	8	*c.832* *A* *>* *G, p.Thr278Ala*	Hemizygous
	10-III:2	Male	Unaffected	8	*c.832* *A* *>* *G, p.Thr278Ala*	Hemizygous
	10-III:3	Female	Carrier	8	*c.832* *A* *>* *G, p.Thr278Ala*	Heterozygous
	10-II:4	Female	Carrier	8	*c.832* *A* *>* *G, p.Thr278Ala*	Heterozygous
11	11-III:1	Female	Affected	7	*c.628* *G* *>* *T, p.Glu210Ter*	Heterozygous
	11-II:2	Male	Unaffected		ND	
	11-II:3	Female	Unaffected		ND	
12	12-III:1	Male	Affected	7	*c.679* *C* *>* *T, p.Gln227Ter*	Hemizygous
	12-II:4	Female	Carrier	7	*c.679* *C* *>* *T, p.Gln227Ter*	Heterozygous
	12-II:2	Male	Unaffected		ND	
	12-III:2	Male	Unaffected		ND	
13	13-III:3	Male	Affected	5	*c.389_390delTT, p.Phe130SerfsTer4*	Hemizygous

*RPGR* transcript ID: NM_001034853.1

*ND* not detected

Novel variants are shown in italic

Whole-exome sequencing with targeted analysis for retinal disease-causing genes on RetNET (https://sph.uth.edu/retnet/) was performed in 18 affected, 9 carriers, and 14 unaffected subjects from 13 families

Sequence variant nomenclature was obrained according to the guidelines of the Human Genome Variation Society by using Mutalyzer (https://mutalyzer.nl/)

Thirteen *RPGR* variants were identified; c.3399delG, p.Pro1134HisfsTer18; c.3308_3309delAT, p.Tyr1103SerfsTer7; c.3178_3179delGA, p.Glu1060ArgfsTer18; c.3104_3105delAG, p.Glu1035GlyfsTer43; c.3092delA, p.Glu1031GlyfsTer58; c.2625dupA, p.Gly876ArgfsTer203; c.2236_2237delGA, p.Glu746ArgfsTer23; c.1693C > T, p.Gln565Ter; c.1070 G > A, p.Gly357Asp; c.832 A > G, p.Thr278Ala; c.628 G > T, p.Glu210Ter; c.679 C > T, p.Gln227Ter; and c.389_390delTT, p.Phe130SerfsTer4. Twelve variants were detected by WES with target analysis of the retinal disease-associated genes. One variant was found by specific direct sequencing for RPGR-ORF15 (p.Gly876ArgfsTer203).

There are eight frameshift, three nonsense, and two missense variants. Five variants have been previously reported^18,26,29,32^. Eight variants have never been reported, including three frameshift, three nonsense, and two missense variants: p.Pro1134HisfsTer18, p.Glu1035GlyfsTer43, p.Phe130SerfsTer4, p.Gln565Ter, p.Glu210Ter, p.Gln227Ter, p.Gly357Asp, and p.Thr278Ala.

### In silico molecular genetic analysis

The detailed results of in silico molecular genetic analyses for the 13 detected *RPGR* variants are presented in Supplementary Table 2.

Seven frameshift variants were located in ORF15. Six variants, including three nonsense, two missense, and one frameshift variant, were in exons 5, 7, 8, 10, and 14, and all six variants except for one frameshift variant (p. Gly876ArgfsTer203) were located in the RCC1-like domain. The allele frequency for one variant (p. Glu1060ArgfsTer18) in the general population was 0.001134% in the GnomAD database. None of the 13 detected *RPGR* variants were found in the Japanese general population of the HGVD and iJGVD databases.

General prediction, functional prediction, and conservation were assessed for the 13 variants, and pathogenicity classification according to the ACMG guidelines was pathogenic for eight variants, including six frameshift (p.Glu746ArgfsTer23, p.Glu1031GlyfsTer58, p.Glu1035GlyfsTer43, p.Glu1060ArgfsTer18, p.Tyr1103SerfsTer7, and p.Pro1134HisfsTer18) and two nonsense variants (p.Glu210Ter and p.Gln565Ter), likely pathogenic for three variants including two frameshift (p.Phe130SerfsTer4 and p.Gly876ArgfsTer203) and one nonsense variant (p. Gln227Ter), and uncertain significance for two missense variants (p.Gly357Asp and p.Thr278Ala).

Two missense variants (p. Gly357Asp and p. Thr278Ala) with uncertain significance were found in two probable XL families (Families 9, 10), and no other candidate variants associated with RP were detected in either of these families.

Overall, 13 disease-causing variants in the *RPGR* gene were ascertained in eight families with RP, and five families with CORD. Together with the clinical features of affected subjects and the model of inheritance in the pedigree, 13 disease-causing variants in the *RPGR* gene were determined.

### Genotype–phenotype association

The locations of variants for two phenotype subgroups were investigated. All variants in eight families with RP were located in exons 1–14 and the 5′ end of ORF15 (< amino acid 950). All variants in five families with CORD were located at the 3′ end of ORF15 (> amino acid 950). A significant genotype–phenotype association between the phenotype subgroup and the position of detected variants was revealed.

## Discussion

The clinical and genetic characteristics of *RPGR*-RD were illustrated in a nationwide cohort of 18 affected and 14 unaffected individuals and 9 carriers from 13 Japanese families with *RPGR*-RD, detecting 13 variants including 8 novel variants. There were eight families with RP and five families with CORD, which was associated with the position of *RPGR* variants.

To the best of our knowledge, this study reports the largest cohort of *RPGR*-RD in the Asian population. *RPGR*-RD accounts for 66.7% of molecularly confirmed XLRP (12 families; RPGR-8 families, RP2-4 families) in the JEGC cohort. All of molecularly confirmed XLCORD were *RPGR*-RD (RPGR-5 families). *RPGR*-RD accounts for 8.1% of 148 families with molecularly confirmed RP in total, and accounts for 4.8% of 105 families with molecularly confirmed CORD and allied disorders. The prevalence of *RPGR*-RD was similar to that of European cohorts^3,48,49^, although *CACNA1F* responsible for XL incomplete congenital night blindness (incomplete type of Miyake’s classification; OMIM: 300071)^50,51^ was not included as CORD in the JEGC cohort.

Out of 13 detected *RPGR* variants detected in this study, five variants were located within the RCC1-like domain (5/13, 38.5%), and seven were within the ORF15 domain (7/13, 53.8%). The proportion of ORF15 variants in the present cohort was slightly lower than that of the North American population (66%, reported by Sharon et al.)^27^.

In this study, five previously reported *RPGR* variants were identified in five families (Families 2, 3, 5 -CORD; Families 6, 7 -RP). Three of the five previously reported variants in the present cohort were reported in European cases with RP (p. Glu746ArgfsTer23, p.Gly876ArgfsTer203, and p.Glu1031GlyfsTer58)^18,26,33^. The other two variants were reported in European cases with CORD (p.Glu1060ArgfsTer18 and p.Tyr1103SerfsTer7)^29,32^. Four of the five families (4/5, 80%) in the present cohort showed a concordant phenotype with previous reports (Families 2, 3, 6, 7). In that study, the clinical effect of these four variants was confirmed in the Japanese population. One family with CORD had a discordant phenotype with a previous report (Family 5, p.Glu1031GlyfsTer58). Given the severely affected retinal findings in two affected males in Family 5, an advanced stage of this phenotype could be described as “RP”.

Eight *RPGR* variants were reported first in this study, including three frameshift (p.Phe130SerfsTer4, p.Glu1035GlyfsTer43, and p.Pro1134HisfsTer18), three nonsense (p.Glu210Ter, p.Gln227Ter, and p.Gln565Ter), and two missense variants (p.Thr278Ala and p.Gly357Asp). A high proportion of novel variants (8/13, 61.5%) was revealed in the Japanese cohort, which suggests a distinct genetic background for RPGR in the Japanese population compared with the European population.

In silico analysis for eight novel variants predicted pathogenic effects in four variants (p. Glu210Ter, p.Gln565Ter, p.Glu1035GlyfsTer43, and p.Pro1134HisfsTer18), a likely pathogenic effect in two variants (p.Gln227Ter and p.Phe130SerfsTer4), and variants of uncertain significance in two variants (p.Thr278Ala and p.Gly357Asp). These two missense variants (p.Gly357Asp and p.Thr278Ala) were located within the RCC1-like domain, where other missense disease-causing *RPGR* variants are frequently found^37^. The pathogenicity of these two missense variants is uncertain, although some association with nucleocytoplasmic transport and regulation of cell-division processing may be anticipated^16,17^.

A significant association between genotype and phenotype was revealed in this study. Variants located in exons 1–14 and the 5′ end of ORF15 caused RP, and variants at the 3′ end of ORF15 caused CORD. This finding was consistent with previous reports in the European population^3,29,30^. This fact supports the prediction of the natural history of *RPGR*-RD in counseling patients. Notably, the mechanism underlying this genotype–phenotype association has not been clarified.

There are limitations to this study. The selection bias related to the disease severity should be inherent, since it is uncommon for genetically affected subjects with good vision to visit clinics/hospitals. This study is a cross-sectional retrospective study; thus, longitudinal natural history studies in a larger cohort could provide more accurate information for disease progression of *RPGR*-RD. In addition, the molecular mechanisms of disease causation for some novel and previously reported variants are not yet known; therefore, further functional analysis could determine the disease causation of each variant.

In conclusion, the phenotypic and genotypic features of *RPGR*-RD were documented first in a large cohort of Japanese populations. A broad spectrum of phenotypic and genotypic findings was determined, revealing a considerable genotype–phenotype association. This evidence should be helpful in monitoring and counseling patients and in selecting patients for future therapeutic trials such as gene replacement therapy.

## Supplementary information

Supplemental Figure 1

Supplemental Figure 2

Supplementary Table 1

Supplementary Table 2
