# Correction to: Clinical and genetic characteristics of 14 patients from 13 Japanese families with *RPGR*-associated retinal disorder: report of eight novel variants

**DOI:** 10.1038/s41439-019-0086-2

**Published:** 2020-02-10

**Authors:** Go Mawatari, Kaoru Fujinami, Xiao Liu, Lizhu Yang, Yu Fujinami-Yokokawa, Shiori Komori, Shinji Ueno, Hiroko Terasaki, Satoshi Katagiri, Takaaki Hayashi, Kazuki Kuniyoshi, Yozo Miyake, Kazushige Tsunoda, Kazutoshi Yoshitake, Takeshi Iwata, Nobuhisa Nao-i, on behalf of the JEGC study group

**Affiliations:** 1 Department of Ophthalmology, Faculty of Medicine, University of Miyazaki, Kiyotake, Miyazaki Japan; 2 Laboratory of Visual Physiology, Division of Vision Research, National Institute of Sensory Organs, National Hospital Organization Tokyo Medical Center, Meguro-ku, Tokyo, Japan; 3 Department of Ophthalmology, Keio University School of Medicine, Tokyo, Japan; 4 UCL Institute of Ophthalmology, London, UK; 5 Moorfields Eye Hospital, London, UK; 6 Southwest Hospital/Southwest Eye Hospital, Third Military Medical University, Chongqing, China; 7 Graduate School of Health Management, Keio University, Tokyo, Japan; 8 Division of Public Health, Yokokawa Clinic, Suita, Osaka Japan; 9 Department of Ophthalmology, Nagoya University Graduate School of Medicine, Showa-ku, Nagoya, Aichi Japan; 10 Department of Ophthalmology, The Jikei University School of Medicine, Nishi-Shimbashi, Minato-ku, Tokyo, Japan; 11 Department of Ophthalmology, Kinki University Faculty of Medicine, Osaka-Sayama City, Osaka Japan; 12 Kobe Eye Center, Next Vision, Kobe, Hyogo Japan; 13 Division of Molecular and Cellular Biology, National Institute of Sensory Organs, National Hospital Organization Tokyo Medical Center, Meguro-ku, Tokyo, Japan

**Correction to: Human Genome Variation**

10.1038/s41439-019-0065-7, published online 2 August 2019

After online publication of this article, the authors noticed an error in the Author name, Results and Discussion section, as well as in Fig. 1 and Table 2.

**Figure 1:**
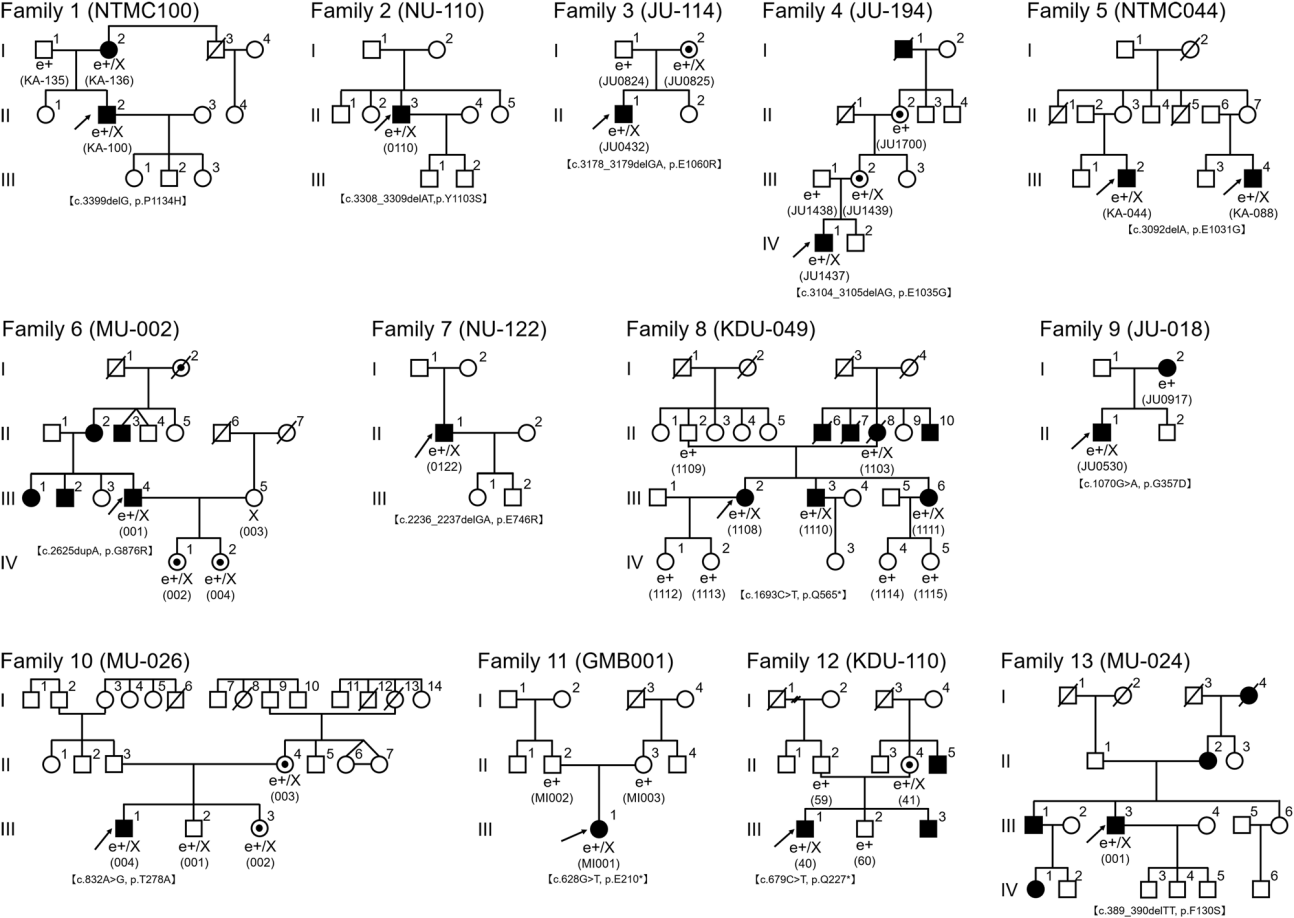
Fig. 1

**Table 2:** Summary of detected variants of 19 affected and 22 unaffected individuals from 13 families with RPGR-associated retinal disorder.

Fmily No.	Patient No.	Gender	Affected/unaffected	Exon	Nucleotide and amino acid changes	State
1	1-II:2	Male	Affected	15	*c.3399delG, p.Pro1134HisfsTer18*	Hemizygous
	1-I:1	Male	Unaffected		ND	
	1-I:2	Female	Affected	15	*c.3399delG, p.Pro1134HisfsTer18*	Heterozygous
2	2-II:3	Male	Affected	15	c.3308_3309delAT, p.Tyr1103SerfsTer7	Hemizygous
3	3-II:1	Male	Affected	15	c.3178_3179delGA, p.Glu1060ArgfsTer18	Hemizygous
	3-I:1	Male	Unaffected		ND	
	3-I:2	Female	Unaffected	15	c.3178_3179delGA, p.Glu1060ArgfsTer18	Heterozygous
4	4-IV:1	Male	Affected	15	*c.3104_3105delAG, p.Glu1035GlyfsTer43*	Hemizygous
	4-III:1	Male	Unaffected		ND	
	4-III:2	Female	Unaffected	15	*c.3104_3105delAG, p.Glu1035GlyfsTer43*	Heterozygous
	4-II:2	Female	Unaffected		ND	
5	5-III:2	Male	Affected	15	c.3092delA, p.Glu1031GlyfsTer58	Hemizygous
	5-III:4	Male	Affected	15	c.3092delA, p.Glu1031GlyfsTer58	Hemizygous
6	6-III:4	Male	Affected	15	c.2625dupA, p.Gly876ArgfsTer203	Hemizygous
	6-IV:1	Female	Unaffected	15	c.2625dupA, p.Gly876ArgfsTer203	Heterozygous
	6-III:5	Female	Unaffected		ND	
	6-IV:2	Female	Unaffected	15	c.2625dupA, p.Gly876ArgfsTer203	Heterozygous
7	7-II:1	Male	Affected	15	c.2236_2237delGA, p.Glu746ArgfsTer23	Hemizygous
8	8-III:2	Female	Affected	14	*c.1693C>T, p.Gln565Ter*	Heterozygous
	8-II:8	Female	Affected	14	*c.1693C>T, p.Gln565Ter*	Heterozygous
	8-II:2	Male	Unaffected		ND	
	8-III:3	Male	Affected	14	*c.1693C>T, p.Gln565Ter*	Hemizygous
	8-III:6	Female	Affected	14	*c.1693C>T, p.Gln565Ter*	Heterozygous
	8-IV:1	Female	Unaffected		ND	
	8-IV:2	Female	Unaffected		ND	
	8-IV:4	Female	Unaffected		ND	
	8-IV:5	Female	Unaffected		ND	
9	9-II:1	Male	Affected	10	*c.1070G>A, p.Gly357Asp*	Hemizygous
	9-I:2	Female	Affected		ND	
10	10-III:1	Male	Affected	8	*c.832A>G, p.Thr278Ala*	Hemizygous
	10-III:2	Male	Unaffected	8	*c.832A>G, p.Thr278Ala*	Hemizygous
	10-III:3	Female	Unaffected	8	*c.832A>G, p.Thr278Ala*	Heterozygous
	10-II:4	Female	Unaffected	8	*c.832A>G, p.Thr278Ala*	Heterozygous
11	11-III:1	Female	Affected	7	*c.628G>T, p.Glu210Ter*	Heterozygous
	11-II:2	Male	Unaffected		ND	
	11-II:3	Female	Unaffected		ND	
12	12-III:1	Male	Affected	7	*c.679C>T, p.Gln227Ter*	Hemizygous
	12-II:4	Female	Unaffected	7	*c.679C>T, p.Gln227Ter*	Heterozygous
	12-II:2	Male	Unaffected		ND	
	12-III:2	Male	Unaffected		ND	
13	13-III:3	Male	Affected	5	*c.389_390delTT, p.Phe130SerfsTer4*	Hemizygous

*RPGR* transcript ID: NM_001034853.1. Whole-exome sequencing with targeted analysis for retinal disease-causing genes on RetNET (https://sph.uth.edu/retnet/) was performed in 19 affected and 22 unaffected subjects from 13 families. Sequence Variant Nomenclature was obrained according to the guidelines of the Human Genome Variation Society by using Mutalyzer (https://mutalyzer.nl/).

Novel variants are shown in Italic.

*ND* not detected.

The correct Author name, Results section, Discussion section, Fig. 1, and Table 2 information should have read as follows:

“First, the correct author name is as follows:

Yu Fujinami-Yokokawa.

Second, the number of affected patients (14) refers to the number of probands as mentioned in the title and Results section. The number of carrier patients (7) refers to the carriers regardless of the symptoms as presented in the Results section.

Third, the correct notation symbol for “Family 9-I:2” in Fig. 1 is a solid circle.

All the carriers except Family 9-I:2 were “unaffected” as presented in Table 2. Therefore, the number of affected and unaffected patients were 19 and 22, respectively. The 19 patients affected in Table 2 included 14 probands, 4 carriers with symptoms, and III-3 of Family 8.

Last, the correct notation of the first sentence in the Discussion section is as follows:

The clinical and genetic characteristics of *RPGR*-RD were illustrated in a nationwide cohort of 15 affected individuals (14 probands and III-3 of Family 8), 14 unaffected individuals, and 12 carriers (4 carriers with symptoms and 8 carriers without symptoms) from 13 Japanese families with *RPGR*-RD, detecting 13 variants including 8 novel variants.”

The authors apologize for the inconvenience caused.

